# Microstructural Influences on Fracture at Prior Austenite Grain Boundaries in Dual-Phase Steels

**DOI:** 10.3390/ma12223687

**Published:** 2019-11-08

**Authors:** Luv Sharma, Ron H. J. Peerlings, Marc G. D. Geers, Franz Roters

**Affiliations:** 1 Department of Mechanical Engineering, Eindhoven University of Technology, P.O. Box 513, 5600 MB Eindhoven, The Netherlands; r.h.j.peerlings@tue.nl (R.H.J.P.); m.g.d.geers@tue.nl (M.G.D.G.); 2 Microstructure Physics and Alloy Design, Max-Planck-Institut für Eisenforschung, Max-Planck-Str. 1, 40237 Düsseldorf, Germany; f.roters@mpie.de

**Keywords:** DP steels, prior austenite grain boundaries, martensite variants

## Abstract

Dual phase (DP) steels provide good strength and ductility properties. Nevertheless, their forming capability is limited due to the damage characteristics of their constituting microstructural phases and interfaces. In this work, a specific type of interface is analysed, i.e., prior austenite grain boundaries (PAGBs). In the literature, prior austenite grain boundary fracture has been reported as an important damage mechanism of DP-steels. The influence of the morphology of phase boundaries near the PAGB and the role of the martensite substructure in the vicinity of a PAGB on damage initiation is analysed. The experimentally observed preferred sites of crack nucleation along the PAGB are assessed and clarified. A finite strain rate dependent crystal plasticity model accounting for the anisotropic elasto-plasticity of martensite (and also ferrite) was applied to an idealized volume element approximating a typical small-scale PAGB microstructure. The boundary value problem is solved using a fast Fourier transform (FFT) based spectral solver. The role of crystallography and geometrical features within the volume element is studied using simulations. Results are discussed considering possibly dominant regimes of elasticity and plasticity.

## 1. Introduction

A combination of martensite and ferrite, in an optimal proportion, lends dual phase (DP) steel an adequate balance between high strength and ductility. At the same time, these phases—due to the contrast in their micromechanical responses—create microstructurally heterogeneous stress and strain fields. Recent computational studies on dual phase steels have focused on exploring the causes of strain partitioning among ferrite and martensite, for example [1]. Martensite itself has been a topic of research addressing its complex micromechanical behavior. One reason of the complexity is the martensite internal structure which can have a detailed hierarchy, as frequently reported in the experimental literature. Refs. [2,3] studied the crystallographic relationship between these hierarchical features. Ref. [2] also reports the dependence of relative feature sizes on the carbon content, or more generally their chemistry, which is another important aspect of steels. The crystallography of martensite is related to the prior austenite grain orientation and has been quantified using specific orientation relationships, Kurdjumov–Sachs (KS) [4], Nishiyama–Wassermann (NW) [5], etc. The fineness of these crystallographic components entails a high concentration of interfaces in the microstructure. Motivated by the possible relevance of size effects in the fine microstructure of tempered martensite, a size dependent crystal plasticity model was used in [6] to explore the effect of martensite block width and misorientation across the blocks. Identification of the microstructural features at such small scales (≈nm–μm) has been difficult, whereby [7] identified the existence of interphases like retained-austenite. The presence of such phases has allowed the ability to explain the small-scale ductile behavior of martensite, see [8]. Ref. [9] used a laminate rule of mixture (lath martensite and austenite) in a local crystal plasticity setting to illustrate its significant effect on the micromechanical response despite its low volume fraction.

Recent experimental work [10] has identified the prior austenite grain boundaries (PAGBs) within the martensite islands as preferable sites for damage nucleation. This has been attributed to a combination of sharp curvature of the phase boundaries near their intersection with the PAGBs and embrittlement of the PAGBs due to chemical segregation, see [10] and references therein. Interestingly, some PAGBs have been reported to damage away from the phase boundaries. In first part of this contribution, we seek to understand the possible reasons for the preferential crack nucleation along PAGBs.

A subsequent work [11], attempts to correlate the damage at PAGBs with the orientation relationship of the martensite crystals nucleating in adjacent prior austenite grains. Motivated by the compatibility of transformation strains of the martensite crystals, a “double KS” orientation relationship was suggested for damage resistant PAGB segments. In a previous work [12], this compatibility is discussed in terms of mismatch in transformation strains, which is being referred to as transmission of shape strain. This suggestion seems related to the ideas of coherent transformation. Refs. [13,14] discussed the role of coherency in transformation-related orientation relationships in providing martensite interfaces that have low misalignment of certain (active) slip systems and similarly of the cleavage systems. In the second part of the present work, we make use of the knowledge of the eigen strains associated with the transformation process. This allows to explore the role of coherency of transformation strains, to gain further insight into the crystallography that makes prior austenite grain boundaries more (or less) damage resistant.

To this effect, an idealization of a small region containing a PAGB intersecting a martensite–ferrite phase boundary is considered. The complexity of the substructure is reduced by assuming uniquely identified regions of martensite variants in the two prior austenite grains. In particular, a “critical” microstructure is sought after, which leads to higher stresses at PAGB and away from the phase boundaries. A fast Fourier transform (FFT) based spectral solver is used for the solution of the micromechanical boundary value problem. The stress distribution is analysed by comparing it with possible heterogeneous fracture property fields, where the heterogeneity may be related to the local orientation of martensite variants, their piecewise misorientation and possibly also chemical segregation.

The remainder of this paper is as follows. In Section 2, the crystal plasticity model and the austenite–martensite orientation relationship are discussed. In Section 3, results of the simulations are presented and analysed. The paper closes with a brief summary and conclusion in Section 4.

## 2. Material Model and Crystallography

Here, the material model used to describe the micromechanical response of martensite and ferrite is discussed first. The crystallography (specific to martensite crystals) used in the material model and the transformation strain analysis is discussed next.

### 2.1. Material Model

The micromechanical responses of martensite and ferrite are anisotropic. Their deformation kinematics as considered in this work follows a finite strain description. The total deformation gradient **F** is decomposed multiplicatively as $F=F_{e}\cdot F_{p}$, where $F_{e}$ and $F_{p}$ are the elastic and plastic deformation gradients.

The elasticity of the ferrite and martensite, which are both body centered cubic (BCC) lattice phases, is governed by the elasticity constants $c_{11},c_{12}$ and $c_{44}$, that are used to construct the stiffness tensor ${}^{4}C$. The stress is calculated using the generalized Hooke’s law for elasticity:

(1) $$\begin{array}{c} S={}^{4}C:E_{e}, \end{array}$$

where S is the work conjugate of the Green–Lagrange strain, $E_{e}=\left(F_{e}^{T}\cdot F_{e}-I\right)/2$. **F**${}_{p}$ is calculated using ${\dot{F}}_{p}=L_{p}\cdot F_{p}$, where **L**${}_{p}$ is composed by mapping the slip rates ${\dot{\gamma }}_{\alpha }$ on individual slip-systems α as, $L_{p}={\dot{\gamma }}_{\alpha }\;{\vec{d}}_{\alpha }⊗{\vec{n}}_{\alpha }$. ${\vec{d}}_{\alpha }$ and ${\vec{n}}_{\alpha }$ are the normalized slip direction and glide plane normal of slip system α, respectively. The slip $\gamma_{\alpha }$ on each slip-system α is calculated using the phenomenological power law based rate form as,

(2) $$\begin{array}{c} {\dot{\gamma }}_{\alpha }={\dot{\gamma }}_{0}{\left(\frac{|\tau_{\alpha }|}{g_{\alpha }}\right)}^{\frac{1}{m}}\mathrm{sign}\;\left(\tau_{\alpha }\right), \end{array}$$

where $\tau_{\alpha }$ is the driving stress resolved on the slip-system α. This driving stress works against an evolving slip resistance $g_{\alpha }$. ${\dot{\gamma }}_{0}$ is a constant and *m* is referred to as the rate sensitivity exponent. The evolution of $g_{\alpha }$ from the initial value $g_{0}$ is given by the hardening rule as,

(3) $$\begin{array}{c} {\dot{g}}_{\alpha }=\left|{\dot{\gamma }}_{\beta }\right|h_{0}h_{\alpha \beta }{\left|1-\frac{g_{\alpha }}{g_{\infty }}\right|}^{a}\;\mathrm{sign}\;\left(1-\frac{g_{\alpha }}{g_{\infty }}\right), \end{array}$$

where $h_{0}$ is a hardening constant, *a* is hardening exponent and $h_{\alpha \beta }$ is the slip-system hardening interaction matrix. The subscript α refers to the slip system under consideration and β to all the other systems. $g_{\infty }$ refers to the saturation value of the flow stress.

Both, the primary and secondary slip systems of the body centered cubic (BCC) lattice are considered. The elasto-plasticity model is implemented in DAMASK [15,16].

For such models, the values of the constituting model parameters depend on the exact chemical composition of the material (for example, the carbon content), the scale of observation, loading rate, etc. Accordingly, the values reported and used in the literature may differ significantly. The precise identification of these parameters is not the purpose of this study. Since a unique validation with the experiments is not pursued (and is not possible with the limited information available), the parameters reported in [1] are used and are listed in Table 1.

As a measure of the effective plastic activity at a material point, the total shear can be defined as $T=\int \Sigma |{\dot{\gamma }}_{\alpha }|\;dt$, that is the accumulation of plastic slip on all the slip systems. The stress and strain distributions in the forthcoming analysis are shown in terms of the Cauchy stress component $\sigma_{11}$ and logarithmic strain $\varepsilon_{11}$ components, respectively.

### 2.2. Orientation Relationships in Martensite

Body centered cubic (BCC)/body centered tetragonal (BCT) martensite is formed as a transformation product from parent face centered cubic (FCC) austenite. During this process a specific relationship emerges between the parent and the product lattice. This special relationship implies parallelism between certain planes (and directions) of the parent and product lattices. Some early experimental works tried to identify this relationship. The work of [4] quantified it in the form of Kurdjumov–Sachs (KS) orientation relationships (ORs), while the result of independent works [5,17] is referred to as the Nishiyama–Wassermann (NW) relationship. The experimental observations thereafter report the presence of either of the above mentioned two ORs and sometimes even others. Here, the KS orientation relationship, wherein the 24 different martensite variants follow cubic symmetry, is assumed. Ref. [18] presented a clear way of relating these different martensite variants. Defining the first KS variant ${\mathrm{KS}}_{1}$ having parallelism of (111)‖(011) of a cube oriented (0^o^,0^o^,0^o^) prior austenite, the other variants can be calculated by the application of the rotation operators $P\in P^{24}$ as given in Table A1. $P^{24}$ is the set of all rotations that maps a cube onto itself. The other KS variants can be written as,

(4) $$\begin{array}{c} O_{{\mathrm{KS}}_{i}}=P_{i}\cdot O_{{\mathrm{KS}}_{1}}\cdot P_{i}^{T}\;\;\mathrm{for}\;i\;\mathrm{in}\;\{1,2,3,\ldots 24\}. \end{array}$$

**Figure 1:**
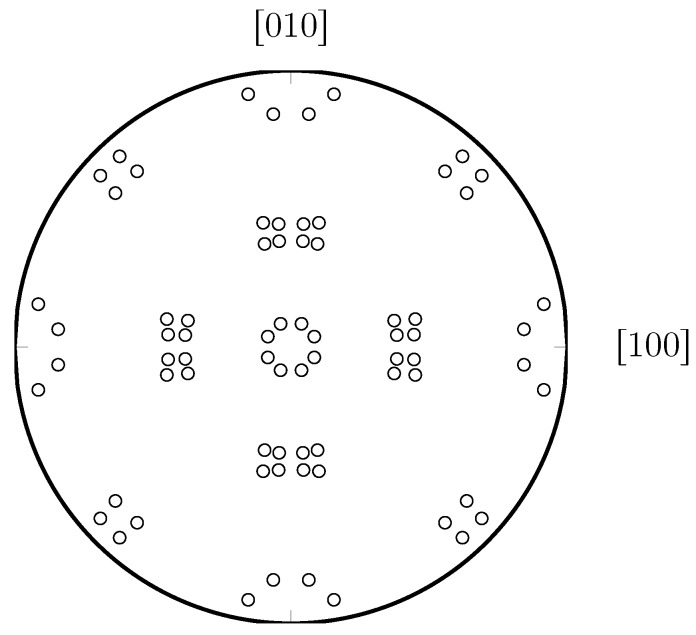
Pole projection of the normals of the {100} family of planes according to Kurdjumov–Sachs (KS) orientation relationships (ORs).

Figure 1 depicts the symmetrical pattern of {100} poles, as also observed in [3,18]. For a differently oriented austenite grain, defined by orientation matrix A, the orientation matrix of the $i^{th}$ KS variant reads

(5) $$\begin{array}{cc} O_{{\mathrm{KS}}_{i}} & =P_{i}\cdot A\cdot O_{{\mathrm{KS}}_{1}}\cdot A^{T}\cdot P_{i}^{T} \end{array}$$

(6) $$\begin{array}{cc} \; & =P_{i}\cdot A\cdot O_{{\mathrm{KS}}_{1}}\cdot {\left(P_{i}\cdot A\right)}^{T}. \end{array}$$

**Figure 2:**
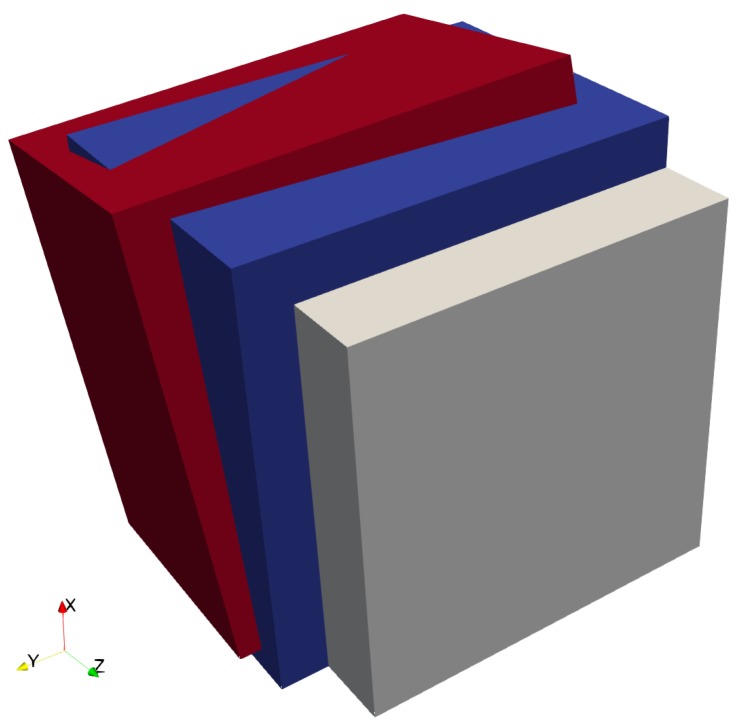
Schematic representation of the Bain strain, the related rotation and the total deformation. The application of $B_{v}\left(=B_{3}\right)$ (grey to blue) applies a compression along the *Z* axis and expansion in the X−Y plane. The subsequent rotation $R_{i}$ (blue to red) is necessary to restore the habit plane as unrotated and the in-plane direction unchanged.

Motivated by the understanding that the Bain mechanism [19] leads to the transformation of austenite to martensite, ref. [18] provides a method to obtain the orientation relationship from transformation strains and vice versa. This procedure is rather straightforward but not well documented in the literature. A lack of a consistent definition of the transformation strains could be a reason. In this work, the following definition of the transformation strain,

(7) $$\begin{array}{c} T_{i}=R_{i}\cdot B_{v} \end{array}$$

is followed and the resulting deformation is shown schematically in Figure 2.

$B_{v}$ is the Bain strain associated with the particular variant with v∈1,2,3 referring to the three possible Bain strains with a different compression axis. For a particular *i* there exists a unique v and the grouping of the variants thus sorted constitutes Bain groups (1,2 and 3). For example, $B_{3}$ has the form,

(8) $$\begin{array}{c} B_{3}=\left(\begin{array}{ccc} \frac{\sqrt{2}a_{\mathrm{BCC}/\mathrm{BCT}}}{a_{\mathrm{FCC}}} & 0 & 0\\ 0 & \frac{\sqrt{2}a_{\mathrm{BCC}/\mathrm{BCT}}}{a_{\mathrm{FCC}}} & 0\\ 0 & 0 & \frac{c_{\mathrm{BCC}/\mathrm{BCT}}}{a_{\mathrm{FCC}}} \end{array}\right), \end{array}$$

with $a_{\mathrm{BCC}/\mathrm{BCT}},c_{\mathrm{BCC}/\mathrm{BCT}}$ and $a_{\mathrm{FCC}}$ the lattice parameters of the respective lattices. For a BCC lattice $c_{\mathrm{BCC}}=a_{\mathrm{BCC}}$ otherwise $c_{\mathrm{BCT}}<a_{BCT}$. $R_{i}$ is the rotation assuring that a particular FCC plane and in-plane direction remain unrotated after application of $T_{i}$. The variant-wise transformation strain for the variants of an austenite crystal with orientation matrix A, is given by

(9) $$\begin{array}{cc} T_{i} & =P_{i}\cdot A\cdot T_{1}\cdot {\left(P_{i}\cdot A\right)}^{T}, \end{array}$$

where $T_{1}$ is the deformation gradient tensor corresponding to one of the variants (of a cube oriented prior austenite grain), denoted as i=1, and $P_{i}=I$ for i=1.

## 3. Results and Discussion

### 3.1. Microstructural Volume Element and Loading Conditions

**Figure 3:**
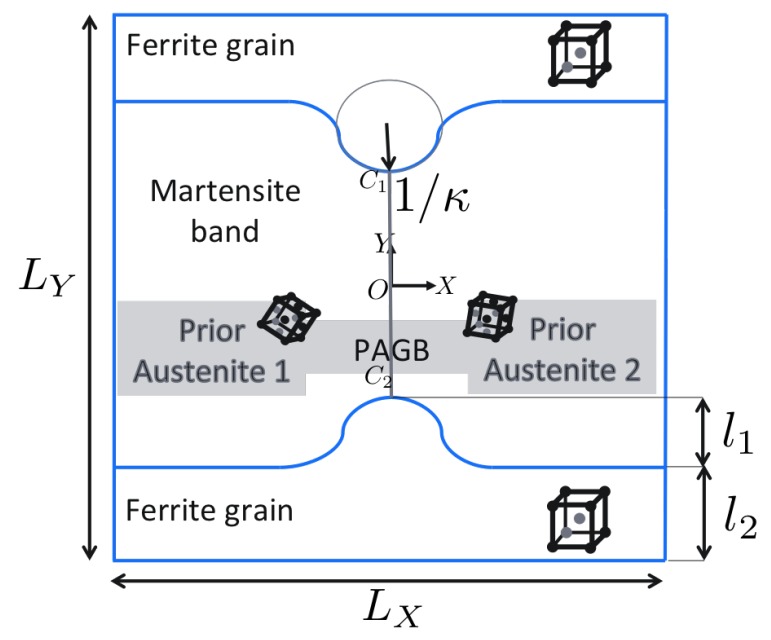
Schematic of the microstructure volume element used in the simulations. It consists of ferrite region of single orientation. The martensite band region transforms from the two prior austenites. The aspect ratio of $L_{X}/L_{Y}=1$ is considered. The origin is at the center and the values of $l_{1}=0.15L_{Y}$ and $l_{2}=0.1L_{Y}$ are considered.

Computational tools that can simulate the mechanics of complex DP-steel microstructures exist, see for example open source code DAMASK. In order to develop a clear understanding, in this contribution we restrict the study to a small region of interest as shown in Figure 3. The ferrite–martensite phase boundary has a curvature κ at the point of intersection with the PAGB. The size of the simulation geometry is denoted by $L_{X}\times L_{Y}$. A columnar grain assumption is made in the out-of-plane direction. It consists of a ferritic region and a martensitic band that emanates from two prior austenite grains separated by the PAGB $C_{1}C_{2}=0.5L_{Y}$. The grains within martensite regions are assumed to have simplified rectilinear shape around the PAGB. The martensite grain orientations are related to the orientation of the prior austenite from which they emanate. Choosing these orientations in further analysis allows us to obtain a substructure with tunable complexity. Since martensite is the major focus of this study, no grain structure within ferrite is considered, i.e., the ferrite region has a single orientation.

The boundary value problem for the mechanical equilibrium of the volume element is solved using an FFT-based spectral solver, which entails periodic boundary condition on the solution field variables of the problem. The resolution of the Fourier grid used is $N_{X}\times N_{Y}=300\times 300$. The loading condition defined in terms of overall rate of deformation gradient $\left({\bar{\dot{F}}}_{}\right)$ and the overall Piola–Kirchhoff stress $\left({\bar{P}}_{}\right)$ is

(10) $$\begin{array}{cccc} {\bar{\dot{F}}}_{} & =\left(\begin{array}{ccc} 10^{-3} & * & 0\\ 0 & * & 0\\ 0 & 0 & 0 \end{array}\right)s^{-1} & ;{\bar{P}}_{} & =\left(\begin{array}{ccc} {}* & 0 & *\\ 0 & *\\ {}* & * \end{array}\right)\mathrm{Pa}. \end{array}$$

Equation (10) implies an applied deformation in X-direction, free contraction in Y-direction and a plane strain assumption in out-of-plane direction. The * refers to an unconstrained deformation/unprescribed stress component.

**Figure 4:**
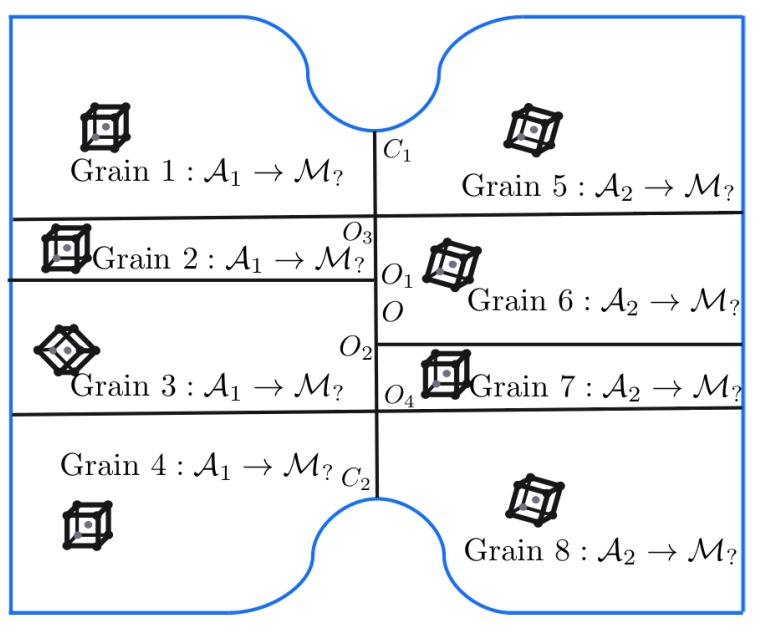
Schematic of the grain structure within martensite band that emanate from the underlying (prior) austenite. The martensite variant, to which the austenite transforms, needs to be identified based on the criticality requirement mentioned earlier. The values of $OO_{1}=OO_{2}=0.06L_{Y}$, $OO_{3}=OO_{4}=0.15L_{Y}$ and $OC_{1}=OC_{2}=0.25L_{Y}$ are used.

Before analysing the micromechanical response of the volume element (Figure 3), single-crystal responses and the effect of phase boundary curvature on individual martensite variants is investigated. These auxiliary studies can help in finding crystals which can be used to render the microstructure critical. The former helps in finding mechanically contrasting crystals which when placed nearby each other in Figure 4, may lead to stress concentration upon loading. The latter helps in selecting crystals which when placed near the curved phase boundaries lead to comparatively low stresses.

### 3.2. Single Crystal Variant Responses to Identify Hard–Soft Variants

In order to consider only critical volume elements, the crystallographic orientations of martensite and ferrite that lead to such situation are needed. In order to achieve that, qualitative input on the hardening behavior of the martensite variants and ferrite crystals is required. Due to the special orientation relationship (Section 2.2) between the martensite variants and the prior austenite grains, it is important to choose the orientation of the latter first. The orientation of the prior austenite grains adjacent to the PAGB may depend on the processing conditions. In order to keep the analysis generic two prior austenite orientations—referred to as $A_{1}$ and $A_{2}$—are arbitrarily chosen and considered for further analysis. Both orientations align the FCC habit planes (111) with the prior austenite grain boundary, but they have a twist misorientation between them. The Euler orientation triplets corresponding to them are given in Table 2.

Corresponding to each prior austenite orientation there are 24 KS-OR related martensite variant orientations, giving rise to numerous possible combinations that can be used to assign the grain orientations in the martensitic substructure. Identifying the martensite variants of each prior austenite as elastically and plastically hard or soft, in the primary loading direction, allows the ability to achieve a critical substructure without considering all mathematically possible combinations. Since anisotropic crystals are being dealt with, which have directional micromechanical response, stress–strain measures need to be defined, on the basis of which they can be rendered hard or soft. For the elastic regime, the slope of $\sigma_{11}$ as a function of $\varepsilon_{11}$ or the directional stiffness $E_{\left[hkl\right]}$ is used. For analysis in the plastic regime, the assessment of hard/soft variants is even more difficult because of the large non-linearity triggered by different model parameters. The hardening parameters, the orientation of active slip systems and the mutual interaction complicate such an analysis. Such an identification may even be transient. Nevertheless, two flow stress values can be used to assess hardness; the von Mises measure of Cauchy stress at low plastic strain T=0.001 and the same at a comparatively high plastic strain T=0.05. Simulations are performed on martensitic single crystals with the orientation of the variants related to the two different prior austenite grains. The loading condition, Equation (10), used here is the same as the one that is intended for the simulation of the full volume element, shown in Figure 3.

**Figure 5:**
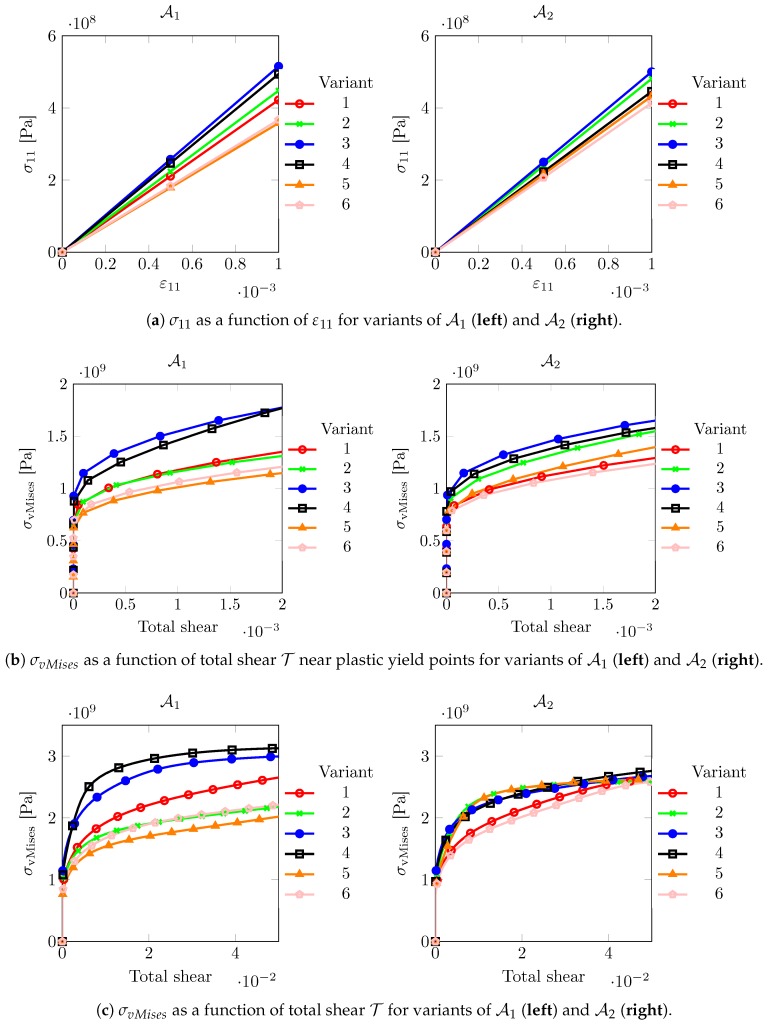
Directional micromechanical responses of homogeneous single crystals with the orientation of martensitic variants related to prior austenite grain $A_{1}$ (**left**) and $A_{2}$ (**right**) by Equation (6) and subjected to the loading given in Equation (10).

Figure 5 shows the directional stress–strain responses in the different regimes as discussed above. It is noted here that only the results of the first six variants, corresponding to the FCC habit planes (111) and $\left(\bar{1}\bar{1}\bar{1}\right)$, are depicted. Due to symmetry, the directional response of the other 18 variants coincides with that of one of the first six variants, thus giving only six or less unique curves in terms of above mentioned stress–strain measures. For prior austenite $A_{1}$, the scatter between the directional micromechanical responses of the variants is a little higher than for $A_{2}$. The variants of both prior austenites show a significant scatter. Thus choosing hard–soft variants from them may provide significant micromechanical contrast in the sub-structural response. Figure 5a shows that the scatter in the directional elastic response of the variants from different prior austenite is different. Figure 5b shows the yield response of different variants. It correlates well with the elastic behavior shown in Figure 5a. At significantly higher plastic strain, Figure 5c, deviations in the trend arise due to differences in hardening behavior.

The result of this analysis is given in Table 3, where the hard and the soft variants, for different strain regimes are listed.

**Figure 6:**
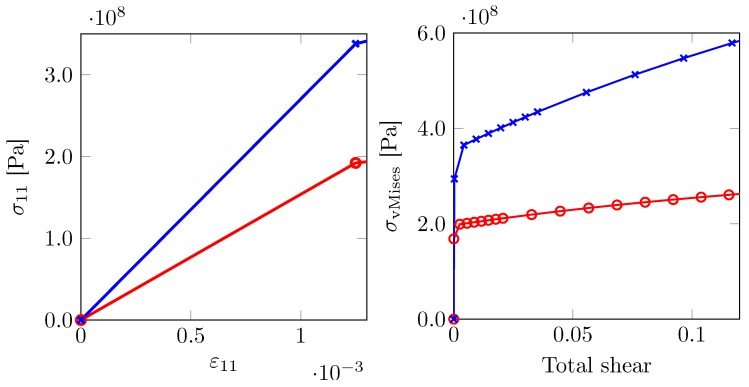
Overall stress–strain response of a single crystal of ferrite. Directionally hard (blue, $F_{\left(45^{0},0^{o},0^{o}\right)})$ and a soft (red, $F_{\left(0^{0},0^{o},0^{o}\right)})$ oriented ferrite.

Similarly, a ferrite single crystal $F_{\left(45^{0},0^{o},0^{o}\right)}$ with orientation given by the Euler triplet set $\left(45^{0},0^{o},0^{o}\right)$ is identified as directionally hard whereas $F_{\left(0^{0},0^{o},0^{o}\right)}$ is identified as soft. Their elasto-plastic responses are shown in Figure 6.

The response of these variants in actual microstructural calculations may significantly change due to microstructural features—phase boundary curvature and martensite substructure near the PAGB (triple junction, distance between triple junctions, active slip-systems, etc.). Note that these single crystal responses only give a qualitative impression of the hardness.

### 3.3. Influence of Phase Boundary Curvature

**Figure 7:**
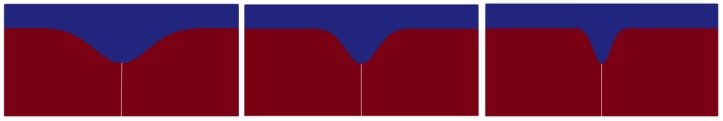
Phase boundaries with different curvature $\kappa_{1}=20l_{1}\;\mu m^{-1}$, $\kappa_{2}=3\kappa_{1}$ and $\kappa_{3}=10\kappa_{1}$. Martensite phase in red, ferrite is in blue and the white line depicts the location where the prior austenite grain boundary (PAGB) would have been if martensite variants of different prior austenite grains were considered.

The curved phase boundaries, although they are not a “notch”, but due to geometry and mechanically contrasting phases may still cause stress concentrations. At the intersection of the curved phase boundaries with the PAGB, the major factors affecting the stress–strain behavior are the curvature κ, the martensite variants adjacent to the tip and the surrounding ferrite. In this section, the analysis is restricted to the earlier identified hard and soft variants resulting from prior austenite $A_{1}$, see Table 3. The effect of phase boundary curvature is analysed using the volume element shown in Figure 3 with different phase boundary curvatures: $\kappa_{1}=20l_{1}$$\mu m^{-1}$, $\kappa_{2}=3\kappa_{1}$ and $\kappa_{3}=10\kappa_{1}$, where $l_{1}$ is given in μm. The zoomed-in section of these volume elements is depicted in Figure 7. The substructure is not populated yet, rather one single orientation (hard or soft) for both the ferrite and martensite region is used. This also implies there is no PAGB or can be considered “pseudo” PAGB with zero misorientation.

In total four combinations are investigated: hard martensite–soft ferrite, hard martensite–hard ferrite, soft martensite–soft ferrite and soft martensite–hard ferrite. Stress and strains ahead of the phase boundary along the path $X=-L_{X}/2N_{X}$ (closest resolved grid points parallel to “pseudo” PAGB at X=0) are analysed at two different instants: when the martensite is in the elastic regime (${\bar{F}}_{11}=1.00125$) and when the martensite has yielded (${\bar{F}}_{11}=1.00875$).

**Figure 8:**
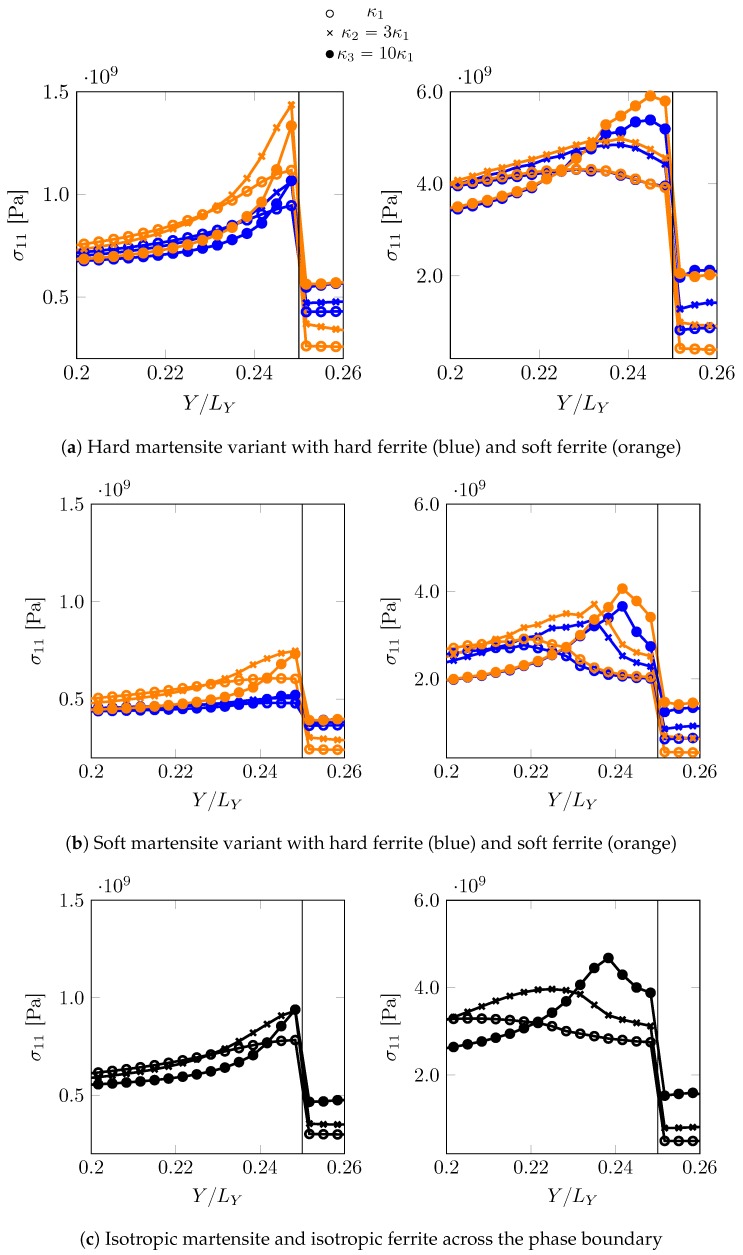
$\sigma_{11}$ distribution profiles (along $X=-L_{X}/2N_{X}$, ahead of the curved phase boundary at $\left(0,0.25L_{Y}\right)$ and extending into the ferrite zone) profiles for different martensite variant-ferrite combinations at two different overall deformation levels, ${\bar{F}}_{11}=1.00125$ (**left** column) and ${\bar{F}}_{11}=1.00875$ (**right** column).

**Figure 9:**
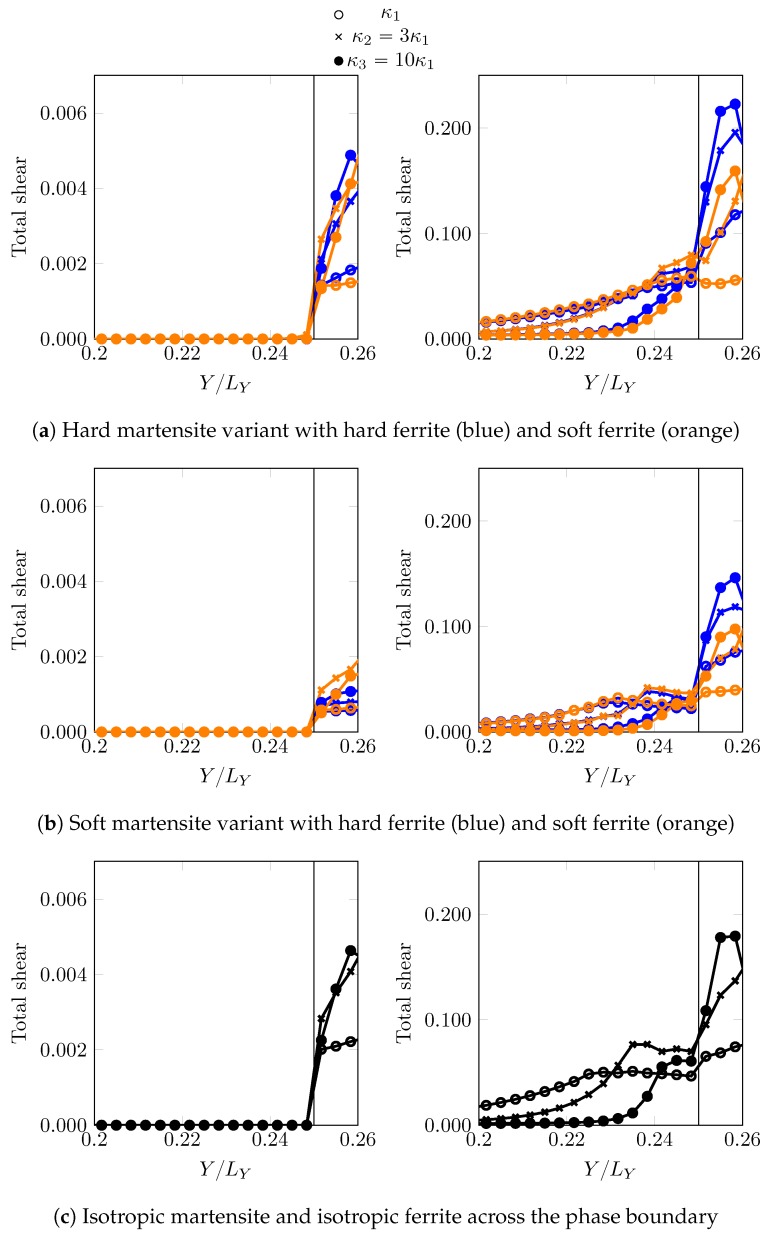
Total shear distribution profiles (along $X=-L_{X}/2N_{X}$, ahead of the curved phase boundary at $\left(0,0.25L_{Y}\right)$ and extending into the ferrite zone) profiles for different martensite variant-ferrite combinations at two different overall deformation levels, ${\bar{F}}_{11}=1.00125$ (**left** column) and ${\bar{F}}_{11}=1.00875$ (**right** column).

The stress $\sigma_{11}$ and total shear T distribution profiles for all these cases are shown in Figure 8 and Figure 9. For the low overall deformation, ${\bar{F}}_{11}=1.00125$, when martensite responds elastically, the peak stress, which is higher for the harder martensite variant, is close to the tip. Having a softer ferrite near the phase boundary, amplifies the peak stresses significantly. Once the region ahead of the curved phase boundary yields (Figure 9), for example at an overall deformation of ${\bar{F}}_{11}=1.00875$, the stress maximum shifts further away. The decay of stress seems characteristic of the phase boundary curvature, implying a sharp drop for sharp curvatures. At low overall deformation, i.e., ${\bar{F}}_{11}=1.00125$, the maximum stress in the martensite ahead of the curvature does not relate linearly with the curvature of the phase boundary. It is comparable for $\kappa_{2}$ and $\kappa_{3}$ in case of $F_{0^{0},0^{o},0^{o}}$, and almost coincides when the adjacent ferrite is $F_{45^{0},0^{o},0^{o}}$. In fact, $\kappa_{2}$ overcomes $\kappa_{3}$ (with sharper curvature) in maximum stress when surrounded by soft ferrite $F_{0^{0},0^{o},0^{o}}$ in Figure 8a. In order to verify whether this is related to the local crystallography or crystallographic differences, a comparison (Figure 8c and Figure 9c) is made with a reference simulation using an isotropic plasticity model parameter (Table 4), which shows a similar trend at ${\bar{F}}_{11}=1.00125$, where the profiles for $\kappa_{2}$ and $\kappa_{3}$ almost coincide. For deformation in the plastic regime of martensite at ${\bar{F}}_{11}=1.00875$, the ambiguity of the relation with the phase boundary curvature vanishes and a systematic trend of higher stress for sharper curvature is observed, which may be related to the size of the yield zone ahead of the curved tip.

In all the cases, the ferrite deforms significantly more than martensite. This is particularly the case when it is more confined, i.e., for higher curvatures κ. This simple comparison highlights the importance of local crystallinity near the curved phase boundary and its possible interplay with the phase boundary morphology (curvature).

### 3.4. Influence of Martensite Substructure

After gaining some clarity on the response of individual crystals (martensite variants and ferrite) and around curved phase boundaries, focus is next put on the other microstructural characteristics. The knowledge of the micromechanical response of the hard–soft martensite variants allows to assign variant orientations to the grains in Figure 4 such that a critical configuration is obtained. The substructure typically consists of triple and even higher order junctions. Triple junctions with surrounding grains of high incompatibility—caused by high micromechanical contrast or mismatch in active slip planes/directions—are preferred regions for stress concentrations. At triple junctions, the orientation of the intersecting interfaces and the hardness of the surrounding variants can be important factors governing stress concentrations. The distance between triple junctions can also influence the stress field in between them.

The knowledge of hard–soft martensite variants, Table 3, can now be used to create junctions that have high mechanical contrast. The curvature of the phase boundary used is $\kappa =\kappa_{2}$. Since a critical configuration requires to avoid high stresses at the phase boundary, the grains (1, 4, 5 and 8) near them should be assigned the orientations of the softer variants corresponding to the respective prior austenite grain. Grains 3 and 6 are assigned a hard variant, while grains 2 and 7 are assigned the soft variant of the corresponding prior austenite based on Table 3, providing four contrasting triple junctions along the PAGB. The ferrite orientation $F_{45^{o},0^{o},0^{o}}$ is considered, as it was found to reduce stress concentrations at the curved phase boundaries in Section 3.3. Two different realizations of the martensite band Figure 4 are considered: $OO_{1}/OO_{2}=1$ (hard bridging martensites across $O_{1}O_{2}$) and $OO_{1}/OO_{2}=-1$ (soft bridging martensites across $O_{1}O_{2}$). Positive and negative values for $O_{1}O_{2}$ refer to situations when the bridging martensite variants are both hard and both soft, respectively.

**Figure 10:**
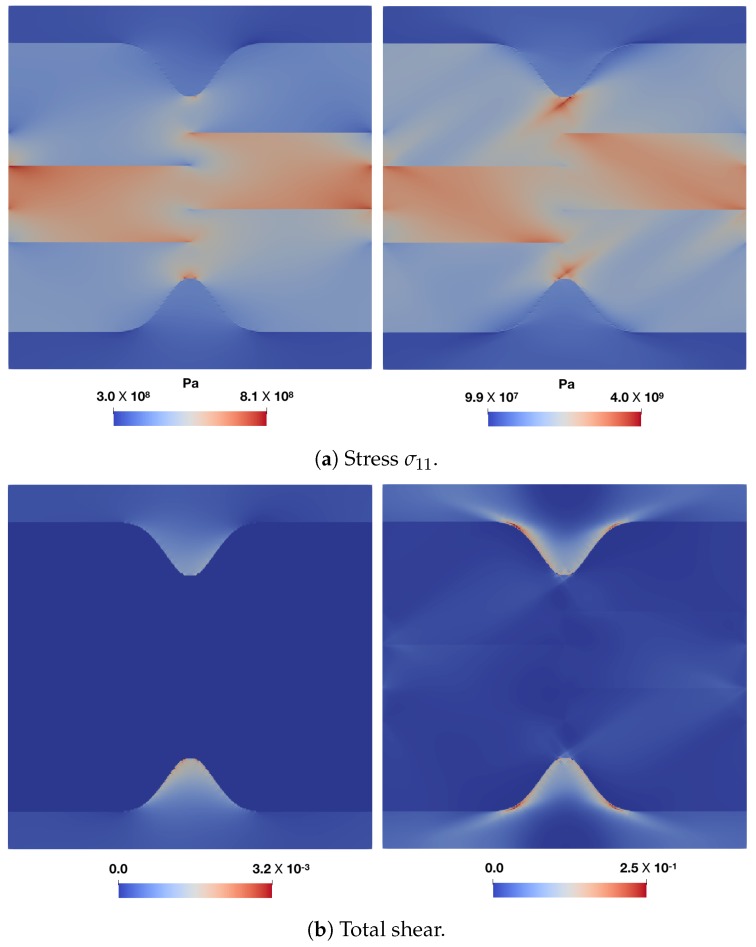
Stress component $\sigma_{11}$ and total accumulated shear T distribution for the volume element (constituting martensite region from prior austenites $A_{1}$ and $A_{2}$), at two different overall deformation levels, ${\bar{F}}_{11}=1.00125$ (**left** column) and ${\bar{F}}_{11}=1.00875$ (**right** column).

In the first set of substructure simulations, the prior austenite orientations $A_{1}$ and $A_{2}$ are considered for the two grains. Figure 10 shows the distribution of the $\sigma_{11}$ component of Cauchy stress and total accumulated shear (T) in the volume element.

**Figure 11:**
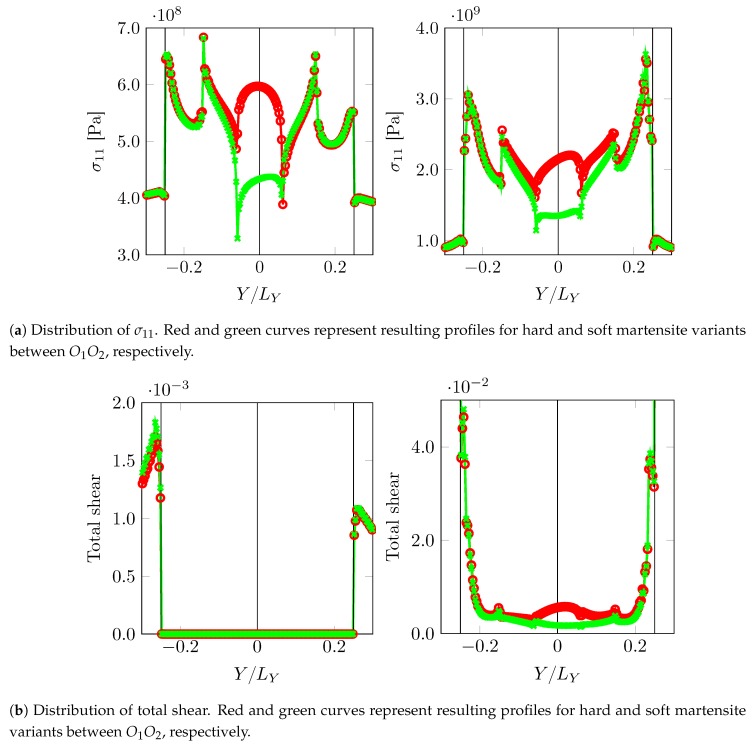
$\sigma_{11}$ and total shear T distribution along path $X=-L_{X}/2N_{X}$ close to PAGB (separating the prior austenite $A_{1}$ and $A_{2}$) at two different overall deformation levels, ${\bar{F}}_{11}=1.00125$ (**left** column) and ${\bar{F}}_{11}=1.00875$ (**right** column).

The curved phase boundaries and the triple junctions are clearly the regions of intensive stress or strain activity. The amount of plastic activity in ferrite is expectedly larger than in martensite. Since the region near the PAGB is in the focus of this work, stress $\sigma_{11}$ and strain (total shear T ) profiles along a parallel line close to it ($X=-L_{X}/2N_{X}$) are plotted in Figure 11 at two different deformation levels ${\bar{F}}_{11}=1.00125$ (left column) and ${\bar{F}}_{11}=1.00875$ (right column).

The decay of stress from the phase boundary curvature tip is similar for all the cases. The stress levels fluctuate significantly at the junctions. In the regime of martensite responding elastically, i.e., at ${\bar{F}}_{11}=1.00125$, the stress around the triple junction ($O_{3},O_{4}$) is almost as high or higher than at the curved phase boundaries. This is the situation that can lead to stress concentration away from the phase boundaries if the critical stress for damage initiation is at a similar level. The regions around the triple junctions ($O_{1},O_{2}$) show a sharp dip in the stresses. This is attributed to the anisotropy of the surrounding crystals deforming to accommodate the incompatibility at these triple junctions. The level of stress between $O_{1}O_{2}$ seems to be governed by the directional hardness of the bridging martensite crystals. Once the plasticity in martensite initiates, stresses at the phase boundary become higher. This is attributed to the modest amount of hardening at the triple junctions along the PAGB. Even at a low plastic strain of ∼0.006 along the PAGB, the transition of higher stress to the curved phase boundary occurs. This transition stage is highly dependent on the local crystallography (misalignment of the active slip systems, etc.).

**Figure 12:**
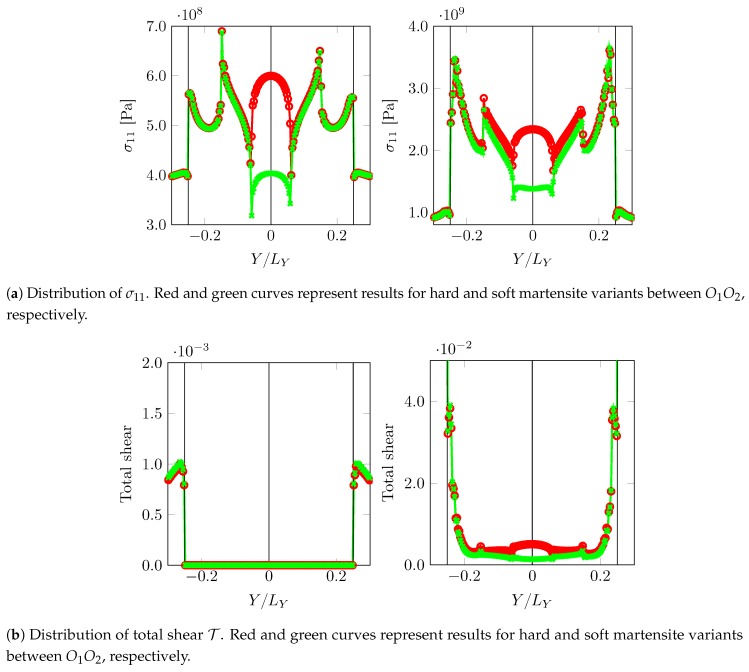
$\sigma_{11}$ and total shear T distribution along path $X=-L_{X}/2N_{X}$ close to zero misorientation PAGB (separating prior austenite $A_{1}$ and $A_{1}$) at two different overall deformation levels, ${\bar{F}}_{11}=1.00125$ (**left** column) and ${\bar{F}}_{11}=1.00875$ (**right** column).

One can conclude that “critical” martensitic substructures can be obtained given a combination of variants providing the required mechanical contrast and crystallography are present in the neighborhood. This situation can also be achieved even when the misorientation of the prior austenite grains is low or even zero as shown in Figure 12. Whether such substructures are plausible as a product of transformation processes is difficult to answer and is the subject of transformation modeling. Knowledge of transformation strains, Equation (9), can still be used to get some insights on their inter-compatibility and is discussed next.

### 3.5. Transformation Strains and Residual Stresses

The previous section assessed the risk for PAGB damage based on the micromechanical response only, given a crystallographic arrangement of martensite variants in a simplified volume element, Figure 3. A crystallography based explanation for experimentally observed stress driven decohesion at different locations along a PAGB was supported. Variant arrangements resulting in less frequently observed decohesion locations, i.e., away from the curved phase boundaries (bounded by martensite phase only), could easily be created. Existence of such substructures in real microstructures is subject to the transformation process (nucleation location, kinetics, transformation-sequencing, etc.).

Transformation modeling can take these aspects into account. For example, phase field based modeling of such transformation processes, refs. [20,21] is inherently capable of capturing the energetic principle based nucleation and growth of martensite variants. The competing kinetics can also be captured using gradient flow models of the Ginzburg–Landau type. Most of the modeling tools are, however, not fully developed for the problem at hand and do not account for all possible nucleating variants corresponding to an orientation relation scheme, with the exception of [22]. Within this restriction, the knowledge of transformation strains associated with individual variants can still be used to extend the understanding gained in the previous section. We propose two further conditions which may support cracking along the PAGB, away from notch like stress concentrators at its intersection with phase boundaries. Both situations relate to the accommodation of transformation strains in the surrounding material.

#### 3.5.1. Strain Incompatibility

The transformation (lattice) strains corresponding to different martensite variants can be strongly anisotropic, see Equation (6). The Bain strain $B_{v}$, Bain strain-related rotation $R_{i}$, austenite orientation A and the symmetry group rotations $P_{i}$, all contribute to this anisotropy. The transformation strains of variants around the PAGB and their mismatch will have to be accommodated by the surrounding material. Nucleating variants tend to minimize this incompatibility, which is also supported by energy minimization principles. Due to the lack of an appropriate model of this type, a theoretical analysis on compatibility of the transformation strains, that relies on the definition of an incompatibility measure for PAGB transformation strains, is presented. Considering a PAGB segment with an interface normal ${\vec{n}}_{PAGB}$, composed of two abutting variants and given the jump in their transformation strain, ∥〚T〛∥, across the interface, the incompatibility can be quantified using Equation (11).

(11) $$\begin{array}{c} X=∥〚T〛\;\times (I-{\vec{n}}_{PAGB}⊗{\vec{n}}_{PAGB})∥. \end{array}$$

The transformation of martensite can initiate either from the interfaces or from inclusions (particles in the bulk of the austenite). In the inclusion case, the presence of a variant at the PAGB is a consequence of the nucleation sequence away from the concerned PAGB. This does not necessarily allow the PAGB segments to be compatible by variant selection as they may have nucleated at other locations of compatibility and grown towards the PAGB considered. Based on earlier work [12], where the habit planes aligned with the PAGB, normal were suggested to be favorable, the investigation is narrowed by only considering transformation of such prior austenite grains. A relationship between the prior austenite grain boundary character and the transformation strain compatibility across ${\vec{n}}_{PAGB}$ is sought. Departing from prior austenite orientations that align a habit plane (111) with the PAGB, various other orientations can be obtained by rotating the crystal about the *X* axis, giving twist misorientation, or about *Z* (where *Z* is out of the plane direction), giving tilt misorientation.

**Figure 13:**
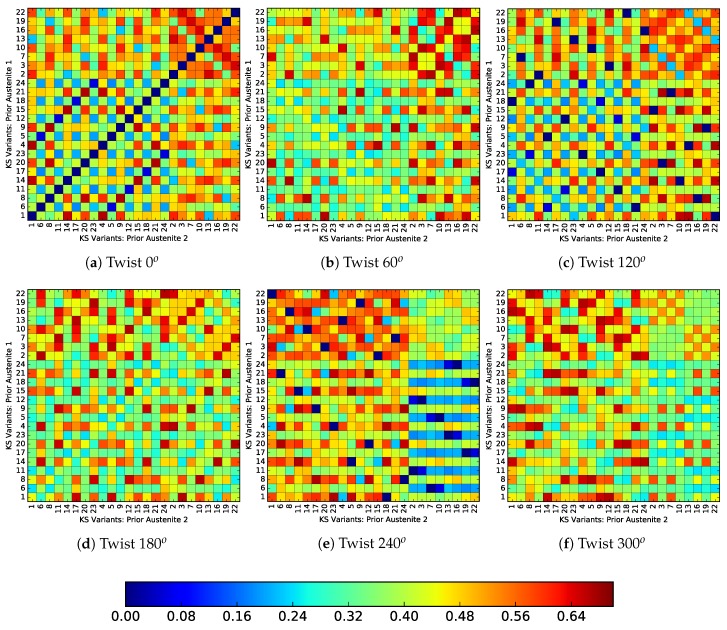
Transformation strain incompatibility for PAGBs with twist misorientation. The variant groups are ordered according to the Bain groups and in the order C first, then the variants of B and A (last), Table A2.

**Figure 14:**
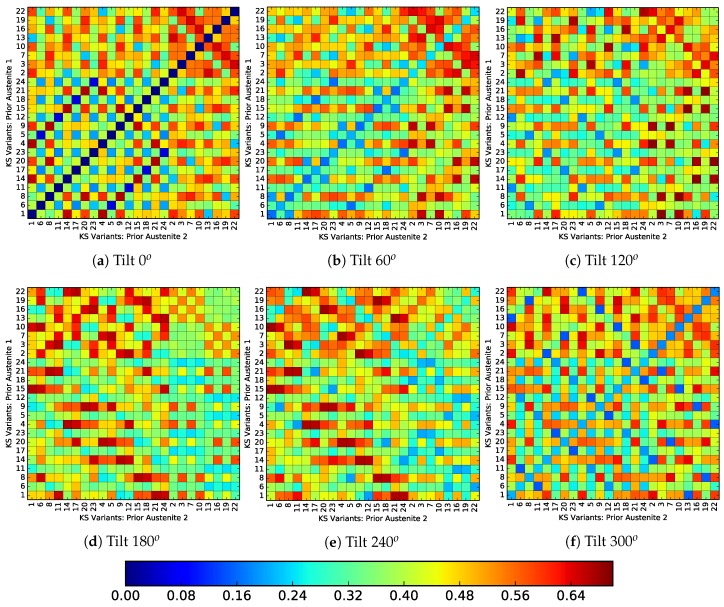
Transformation strain incompatibility for PAGBs with tilt misorientation. The variant groups are ordered according to the Bain groups and in the order C first, then the variants of B and A (last), Table A2.

Figure 13 and Figure 14 show the incompatibility between variants originating from different prior austenite orientations, for a twist misoriented and tilt misoriented PAGB respectively. The horizontal and vertical axes showing the variant numbers of prior austenite 1 and prior austenite 2, respectively. The variant numbers are ordered according to the Bain group, i.e., the eight variants each of group C first, followed by group B and then group A. The color of each voxel represents the incompatibility between the respective variants according to Equation (11). The set of lattice parameters of pure iron ($a_{\mathrm{FCC}}\;=\;0.36313\;\mathrm{nm},a_{\mathrm{BCC}}=0.28974\;\mathrm{nm}$) based on [2] are used. For a PAGB with zero misorientation, Figure 13a and Figure 14a, show that a fully compatible interface is only obtained for identical variants on either side. Combinations resulting in minimum incompatibility X=0 are obtained after each $120^{o}$ twist, Figure 13c,e, which is related to the in-plane direction symmetry. In Figure 13c,e it can be seen that the compatible variants do not necessarily lie within the same Bain group.

It is important to note here that the cleavage planes from the same group have low misorientation [14]. So, if the PAGB were to become weaker on account of cleavage planes aligning with the PAGB, the selection of variants from different groups can enhance the strength. Or, at least render it stronger than when both variants were from the same group, with their low misoriented cleavage planes aligned with the PAGB. On the other hand, the tilt misoriented PAGBs do not show as good compatibility as compared to the twist misoriented ones. In fact no combination of X=0 is observed.

The situation may be even more complex at the triple junctions along the PAGB. The incompatibilities may or may not be accommodated at the PAGBs. Typically, the incompatibilities can be accommodated by plastic straining of the parent austenite grain. This may leave significant amounts of dislocations at the boundaries which may affect their local hardening behavior. The residual stresses thus left may remain in the material after processing, which can be detrimental to the strength of the PAGB. If not accommodated, they may even leave regions of retained FCC phase which may increase the toughness of the PAGB by further transformation upon.

#### 3.5.2. Tension–Compression Residual Stresses

Another interesting aspect of the transformation strain is the tension and compression along different axes. Such transformation strains can leave residual stresses of tensile or compressive nature depending on the orientation of the Bain axis and the constraints induced from the surroundings. It is well possible that compressive stresses near the phase boundaries suppress the damage initiation.

**Figure 15:**
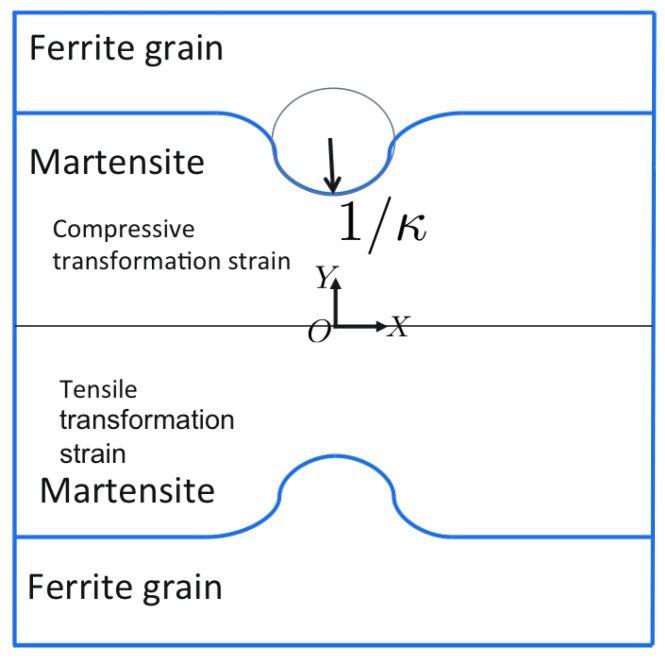
The volume element used for application of transformation strains, Table 5. $\kappa =\kappa_{2}$ is used.

In order to demonstrate this phenomenon, a simulation on a volume element as depicted in Figure 15 is performed. Figure 15 has same overall dimensions as Figure 3. In a first step, transformation strains as given in Table 5 are assigned to different regions.

**Figure 16:**
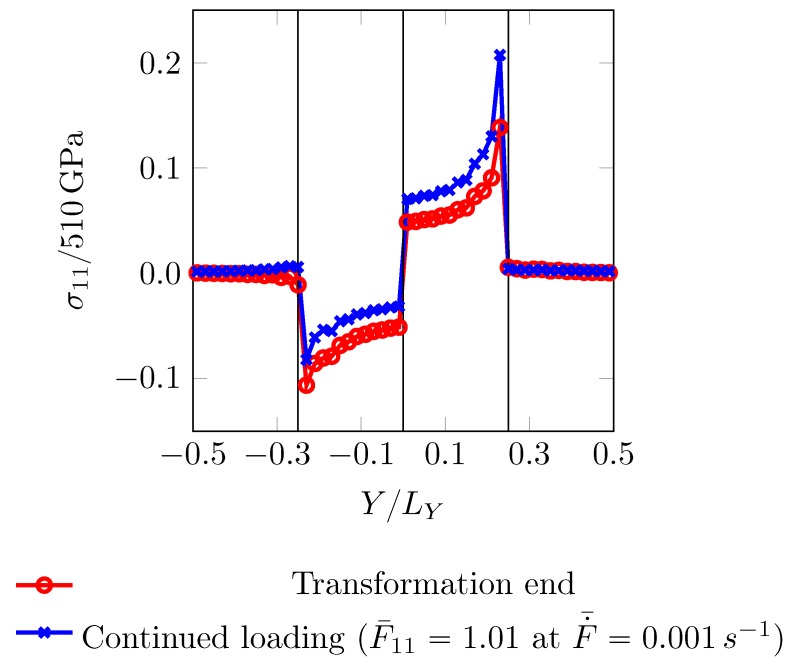
$\sigma_{11}$ profile along the path $X=L_{X}/100$. Normalization is done by the elastic constant $c_{11}$. $\kappa =\kappa_{2}$ is used.

The transformation is assumed to take place in a fully martensitic elastic media. During this transformation a boundary constraint as given by

(12) $$\begin{array}{c} \bar{F}=\left(\begin{array}{ccc} {}* & * & *\\ 0 & * & *\\ 0 & 0 & * \end{array}\right);\;\bar{P}=\left(\begin{array}{ccc} 0 & 0 & 0\\ 0 & 0\\ {}* & 0 \end{array}\right)\mathrm{Pa}, \end{array}$$

is enforced. The martensite accommodating the transformation strains is also constrained by the surrounding ferrite. This process gives rise to residual stresses. Stress component $\sigma_{11}$ along $X=L_{X}/100$ is plotted in Figure 16. From this state, a loading is applied to achieve the final overall deformation state defined by

(13) $$\begin{array}{c} \bar{F}=\left(\begin{array}{ccc} 1.01 & * & 0\\ 0 & * & 0\\ 0 & 0 & 1 \end{array}\right);\;\bar{P}=\left(\begin{array}{ccc} {}* & 0 & *\\ 0 & *\\ {}* & * \end{array}\right)\mathrm{Pa}, \end{array}$$

within 10 s.

$\sigma_{11}$ at the end of this continued loading is also plotted in Figure 16. It can be seen that the initially compressed region loads up slower as compared to the rest and may achieve a critical stress only at higher overall strain level.

## 4. Conclusions

This contribution focused on understanding the phenomenon of martensite cracking in dual-phase steels. The study was particularly motivated by—a less frequent but rather intriguing phenomenon—the observation of PAGB crack nucleation away from the phase boundary. The role of possible mechanistic features that can lead to such behavior has been delineated. The anisotropic elasticity related to the martensite crystal structure triggers stress heterogeneities and concentrations. For a fixed-phase boundary curvature, there exist prior austenite orientations which provide sufficiently high contrasting variants (either elastically, plastically or both) to provide a comparable decohesion stress $\sigma_{11}$ away from the phase boundary even for relatively simple configurations. This possibility exists irrespective of the misorientation between the adjacent prior austenite grains. Even small amounts of plasticity (of the order of 1%) near the PAGB alter the stress concentration location near the phase boundaries.

The second part of the study focused on the possible role of transformation strains and their mismatch in causing residual stresses along PAGBs. Combinations of variants with a small mismatch were identified for given sets of two prior austenite grains. For prior austenite grains with their habit plane parallel to the PAGB, the twist misoriented PAGBs reveal more compatible variants as compared to the tilt misoriented PAGBs. A simplified simulation with tensile–compressive residual stresses was shown to delay the development of tensile stresses (which may lead to decohesion) in regions with compressive residual stresses.

## Figures and Tables

**Table 1:** Phenomenological crystal plasticity model parameters for martensite and ferrite [1].

Parameter	Ferrite	Martensite
Elastic constant, $c_{11}$	233 GPa	417 GPa
Elastic constant, $c_{12}$	135 GPa	242 GPa
Elastic constant, $c_{44}$	118 GPa	211 GPa
Initial slip resistance, $g_{0}$ (${\vec{n}}_{\alpha }\in \left\{110\right\}$ and ${\vec{d}}_{\alpha }\in <111>$)	95 MPa	405 MPa
Initial slip resistance, $g_{0}$ (${\vec{n}}_{\alpha }\in \left\{211\right\}$ and ${\vec{d}}_{\alpha }\in <111>$)	97 MPa	456 MPa
Saturation slip resistance, $g_{\infty }$ (${\vec{n}}_{\alpha }\in \left\{110\right\}$ and ${\vec{d}}_{\alpha }\in <111>$)	222 MPa	872 MPa
Saturation slip resistance, $g_{\infty }$ (${\vec{n}}_{\alpha }\in \left\{211\right\}$ and ${\vec{d}}_{\alpha }\in <111>$)	412 MPa	971 MPa
Hardening constant, $h_{0}$	1 GPa	563 GPa
Self-hardening constant, $h_{\alpha \beta }$ (α=β) self-hardening	1	1
Cross-hardening constant, $h_{\alpha \beta }$ (α≠β) cross-hardening	1.4	1.4
Reference shear rate, ${\dot{\gamma }}_{0}$	$10^{-3}$ s${}^{-1}$	$10^{-3}$ s${}^{-1}$
Rate sensitivity exponent, *m*	0.05	0.05
Hardening exponent, *a*	2.25	2.25

**Table 2:** The Euler angles corresponding to the prior austenite orientations considered.

Prior Austenite	$\varphi_{1}$ $\left({}^{o}\right)$	φ $\left({}^{o}\right)$	$\varphi_{2}$ $\left({}^{o}\right)$
$A_{1}$	−153.4	114.1	140.8
$A_{2}$	−135.0	90.0	144.7

**Table 3:** Identification of variants with hard response (maximum stress) and soft response (minimum stress) in three different loading regimes; elastic, onset of plasticity and significant plasticity for the loading considered.

Prior Austenite	Elastic Regime		$\sigma_{\mathrm{vMises}}{|}_{T≊0.001}$		$\sigma_{\mathrm{vMises}}{|}_{T≊0.05}$	
	Hard Variant	Soft Variant	Hard Variant	Soft Variant	Hard Variant	Soft Variant
$A_{1}$	3	5	3	5	4	5
$A_{2}$	3	6	3	6	4	6

**Table 4:** Taylor factor (*M*) adapted isotropic plasticity ([16]) material parameters for ferrite and martensite.

Parameter	Ferrite	Martensite
Elastic constant, $c_{11}$	286 GPa	510 GPa
Elastic constant, $c_{12}$	121 GPa	216 GPa
Initial shear resistance, $g_{0}$	95.5 MPa	431 MPa
Saturation slip resistance, $g_{\infty }$	317 MPa	922 MPa
Hardening constant, $h_{0}$	1 GPa	563 GPa
Reference shear rate, ${\dot{\gamma }}_{0}$	$10^{-3}$ s${}^{-1}$	$10^{-3}$ s${}^{-1}$
Rate sensitivity exponent, *m*	0.05	0.05
Hardening exponent, *a*	2.25	2.25
Taylor factor, *M*	2.4	2.33

**Table 5:** The transformation strain values applied to the grains in Figure 15.

Region	Transformation Strain
Ferrite	I
Martensite (Y<0)	$\left(\begin{array}{ccc} 1.125 & 0.060 & 0.060\\ -0.074 & 1.111 & 0.134\\ -0.074 & -0.194 & 0.783 \end{array}\right)$
Martensite (Y>0)	$\left(\begin{array}{ccc} 0.783 & 0.074 & 0.194\\ -0.060 & 1.125 & 0.060\\ -0.134 & -0.074 & 1.111 \end{array}\right)$

## Abbreviations

The following abbreviations are used in this manuscript:

DP	Dual phase
PAGB	Prior austenite grain boundary
FFT	Fast Fourier transform
FCC	Face centered cubic
BCC	Body centered cubic
OR	Orientation relationship
KS	Kurdjumov–Sachs
NW	Nishiyama–Wassermann

## Appendix A. Martensite Variant Crystallography

**Table A1:** The rotation operators $P\in P^{24}$ which map a cube onto itself.

$P_{1}=I$	$P_{7}=\hat{R}\left(90^{o},\left[0\bar{1}0\right]\right)$	$P_{13}=\hat{R}\left(180^{o},\left[101\right]\right)$	$P_{19}=\hat{R}\left(90^{o},\left[010\right]\right)$
$P_{2}=\hat{R}\left(180^{o},\left[10\bar{1}\right]\right)$	$P_{8}=\hat{R}\left(180^{o},\left[100\right]\right)$	$P_{14}=\hat{R}\left(180^{o},\left[010\right]\right)$	$P_{20}=\hat{R}\left(180^{o},\left[001\right]\right)$
$P_{3}=\hat{R}\left(120^{o},\left[101\right]\right)$	$P_{9}=\hat{R}\left(180^{o},\left[011\right]\right)$	$P_{15}=\hat{R}\left(90^{o},\left[100\right]\right)$	$P_{21}=\hat{R}\left(90^{o},\left[\bar{1}00\right]\right)$
$P_{4}=\hat{R}\left(180^{o},\left[01\bar{1}\right]\right)$	$P_{10}=\hat{R}\left(120^{o},\left[\bar{1}1\bar{1}\right]\right)$	$P_{16}=\hat{R}\left(120^{o},\left[\bar{1}\bar{1}0\right]\right)$	$P_{22}=\hat{R}\left(120^{o},\left[1\bar{1}\bar{1}\right]\right)$
$P_{5}=\hat{R}\left(-120^{o},\left[111\right]\right)$	$P_{11}=\hat{R}\left(90^{o},\left[011\right]\right)$	$P_{17}=\hat{R}\left(90^{o},\left[00\bar{1}\right]\right)$	$P_{23}=\hat{R}\left(180^{o},\left[\bar{1}\bar{1}0\right]\right)$
$P_{6}=\hat{R}\left(180^{o},\left[1\bar{1}0\right]\right)$	$P_{12}=\hat{R}\left(120^{o},\left[11\bar{1}\right]\right)$	$P_{18}=\hat{R}\left(120^{o},\left[\bar{1}11\right]\right)$	$P_{24}=\hat{R}\left(120^{o},\left[1\bar{1}1\right]\right)$

**Table A2:** The combination of parallel plane normals and parallel in-plane directions in between face centered cubic (FCC) and body centered cubic (BCC) lattices corresponding to the theoretical obtained Kurdjumov–Sachs (KS) orientation relationships.

Variant	Parallel Planes		Parallel Directions		Bain Group
	fcc	bcc	fcc	bcc	
1	(111)	(011)	$\left[10\bar{1}\right]$	$\left[11\bar{1}\right]$	C
2	$\left(\bar{1}\bar{1}\bar{1}\right)$	$\left(\bar{1}\bar{1}0\right)$	$\left[10\bar{1}\right]$	$\left[1\bar{1}\bar{1}\right]$	A
3	(111)	(101)	$\left[\bar{1}10\right]$	$\left[\bar{1}11\right]$	A
4	$\left(\bar{1}\bar{1}\bar{1}\right)$	$\left(0\bar{1}\bar{1}\right)$	$\left[\bar{1}10\right]$	$\left[\bar{1}1\bar{1}\right]$	B
5	(111)	(110)	$\left[0\bar{1}1\right]$	$\left[1\bar{1}1\right]$	B
6	$\left(\bar{1}\bar{1}\bar{1}\right)$	$\left(\bar{1}0\bar{1}\right)$	$\left[0\bar{1}1\right]$	$\left[\bar{1}\bar{1}1\right]$	C
7	$\left(\bar{1}11\right)$	$\left(\bar{1}10\right)$	[101]	[111]	A
8	$\left(1\bar{1}\bar{1}\right)$	$\left(0\bar{1}\bar{1}\right)$	[101]	$\left[1\bar{1}1\right]$	C
9	$\left(\bar{1}11\right)$	(011)	$\left[\bar{1}\bar{1}0\right]$	$\left[\bar{1}\bar{1}1\right]$	B
10	$\left(1\bar{1}\bar{1}\right)$	$\left(10\bar{1}\right)$	$\left[\bar{1}\bar{1}0\right]$	$\left[\bar{1}\bar{1}\bar{1}\right]$	A
11	$\left(\bar{1}11\right)$	$\left(\bar{1}01\right)$	$\left[01\bar{1}\right]$	$\left[\bar{1}1\bar{1}\right]$	C
12	$\left(1\bar{1}\bar{1}\right)$	$\left(1\bar{1}0\right)$	$\left[01\bar{1}\right]$	$\left[11\bar{1}\right]$	B
13	$\left(1\bar{1}1\right)$	$\left(1\bar{1}0\right)$	$\left[\bar{1}01\right]$	$\left[\bar{1}\bar{1}1\right]$	A
14	$\left(\bar{1}1\bar{1}\right)$	$\left(01\bar{1}\right)$	$\left[\bar{1}01\right]$	$\left[\bar{1}11\right]$	C
15	$\left(1\bar{1}1\right)$	$\left(0\bar{1}1\right)$	[110]	[111]	B
16	$\left(\bar{1}1\bar{1}\right)$	$\left(\bar{1}0\bar{1}\right)$	[110]	$\left[11\bar{1}\right]$	A
17	$\left(1\bar{1}1\right)$	(101)	$\left[0\bar{1}\bar{1}\right]$	$\left[1\bar{1}\bar{1}\right]$	C
18	$\left(\bar{1}1\bar{1}\right)$	$\left(\bar{1}10\right)$	$\left[0\bar{1}\bar{1}\right]$	$\left[\bar{1}\bar{1}\bar{1}\right]$	B
19	$\left(11\bar{1}\right)$	(110)	$\left[\bar{1}0\bar{1}\right]$	$\left[\bar{1}1\bar{1}\right]$	A
20	$\left(\bar{1}\bar{1}1\right)$	$\left(0\bar{1}1\right)$	$\left[\bar{1}0\bar{1}\right]$	$\left[\bar{1}\bar{1}\bar{1}\right]$	C
21	$\left(11\bar{1}\right)$	$\left(01\bar{1}\right)$	$\left[1\bar{1}0\right]$	$\left[1\bar{1}\bar{1}\right]$	B
22	$\left(\bar{1}\bar{1}1\right)$	$\left(\bar{1}01\right)$	$\left[1\bar{1}0\right]$	$\left[1\bar{1}1\right]$	A
23	$\left(11\bar{1}\right)$	$\left(10\bar{1}\right)$	[011]	[111]	C
24	$\left(\bar{1}\bar{1}1\right)$	$\left(\bar{1}\bar{1}0\right)$	[011]	$\left[\bar{1}11\right]$	B
